# Endomicroscopic Imaging of COX-2 Activity in Murine Sporadic and Colitis-Associated Colorectal Cancer

**DOI:** 10.1155/2013/250641

**Published:** 2013-01-15

**Authors:** Sebastian Foersch, Clemens Neufert, Markus F. Neurath, Maximilian J. Waldner

**Affiliations:** Department of Medicine 1, University of Erlangen-Nuremberg, Ulmenweg 18, 91054 Erlangen, Germany

## Abstract

Although several studies propose a chemopreventive effect of aspirin for colorectal cancer (CRC) development, the general use of aspirin cannot be recommended due to its adverse side effects. As the protective effect of aspirin has been associated with an increased expression of COX-2, molecular imaging of COX-2, for instance, during confocal endomicroscopy could enable the identification of patients who would possibly benefit from aspirin treatment. In this pilot trial, we used a COX-2-specific fluorescent probe for detection of colitis-associated and sporadic CRC in mice using confocal microscopy. Following the injection of the COX-2 probe into tumor-bearing APCmin mice or mice exposed to the AOM + DSS model of colitis-associated cancer, the tumor-specific upregulation of COX-2 could be validated with in vivo fluorescence imaging. Subsequent confocal imaging of tumor tissue showed an increased number of COX-2 expressing cells when compared to the normal mucosa of healthy controls. COX-2-expression was detectable with subcellular resolution in tumor cells and infiltrating stroma cells. These findings pose a proof of concept and suggest the use of CLE for the detection of COX-2 expression during colorectal cancer surveillance endoscopy. This could improve early detection and stratification of chemoprevention in patients with CRC.

## 1. Introduction

A growing amount of evidence highlights the role of the acetylsalicylate aspirin for the chemoprevention of sporadic colorectal cancer (CRC) [1–4]. Similarly, aminosalicylates such as sulfasalazine, mesalazine, and others have been shown to reduce the risk for colitis-associated colorectal cancer (CAC) in patients with inflammatory bowel disease [5]. In addition, recent data also propose an improved outcome for patients treated with aspirin following the diagnosis of CRC [6]. This is of great importance, as colorectal neoplasia remains one of the leading causes of cancer-related morbidity and mortality in industrialized countries [7].

The effects of aspirin and aminosalicylates are largely attributed to the inhibition of cyclooxygenase-1 (COX-1) and -2. These enzymes convert arachidonic acid to prostaglandin PGH_2_, a precursor molecule for various proinflammatory prostaglandins and eicosanoids. Especially COX-2 has been shown to be responsible for the tumor promoting effects, whereas COX-1 is involved in tissue homeostasis and platelet function [8]. In fact, COX-2 expression is elevated in almost up to 90 percent of sporadic carcinomas and also 40 percent of colonic adenomas, while expression in healthy colonic epithelium remains low [9]. This was also confirmed in experimentally induced colon tumors in rodents [10].

These data propose the use of COX-inhibiting agents for the prevention of sporadic CRC and CAC. It can be achieved by reversible inhibition or irreversible acetylation of COX-1 and/or COX-2. However, the inhibition of COX enzymes is associated with severe side effects in treated patients [11]. In this regard, it would be helpful to quantify COX-2 activity in healthy, inflamed, or dysplastic colonic tissue in order to identify patients that could benefit from the treatment with COX inhibitors as a preventive or therapeutic strategy.

Today, surveillance endoscopy is the gold standard for the prevention of CRC. In addition to conventional endoscopy, technologic advances, such as confocal laser-scanning endomicroscopy (CLE), have recently equipped the gastroenterologist with the astounding possibility of histologic imaging of healthy and altered mucosa during ongoing examination [12, 13]. Importantly, several studies have proposed that CLE can be used for molecular imaging of the large intestine through the use of molecular-targeted fluorescence markers [14, 15]. As the selective visualization of COX-2 expression has recently been explored using a fluorescent probe [16], endomicroscopy of COX-2 expression seems to be a feasible approach.

In this first report, we evaluated the possibility of molecular targeted confocal imaging of COX-2 expression in murine models of colitis-associated and sporadic CRC. This was achieved after systemic injection of a fluorescent COX-2 probe, subsequent in vivo full-body fluorescence imaging and confocal microscopy of unprocessed tissue specimens. In correlation with COX-2 mRNA expression, in-vivo fluorescence imaging, and confocal microscopy showed a strong and specific signal of COX-2 in sporadic and colitis-associated CRC models. As the confocal imaging technique used in this study is also available for endomicroscopy of patients, the analysis of COX-2 expression during CLE could be an applicable and helpful tool for clinical decision-making.

## 2. Materials and Methods

### 2.1. Animals and Models of Sporadic CRC and CAC

Specific pathogen-free C57Bl/6 mice (8–12 weeks old) and APCmin mice were kept in individually ventilated cages and had free access to pellet food and tap-water. CAC was induced in C57Bl/6 mice as previously described [17]. In short, mice were injected with a single dose of the mutagenic agent azoxymethane (AOM) i.p. (7.5 mg/kg bodyweight), followed by three cycles of 2.0% dextran sodium sulfate (DSS) in drinking water and normal drinking water for 1 week. COX-2 expression was analyzed 9 weeks after AOM injection in these animals and at the age of 10 weeks in untreated APCmin mice. These experiments were approved by the State Government of Middle Franconia and conducted according to institutional guidelines.

### 2.2. Imaging of COX-2 Activity

Untreated control mice, AOM + DSS treated mice, and APCmin mice were injected i.p. with a commercially available COX-2 probe (XenoLight RediJect COX-2 probe, Caliper) according to manufacturer guidelines. In vivo full body fluorescence imaging was performed 3 hours following the injection of the probe with a multispectral fluorescence-imaging device (Maestro, Caliper). 4 hours after the injection of the COX-2 probe, mice were sacrificed and healthy, inflamed or tumor tissue was dissected and kept in PBS for immediate confocal imaging without fixation of the tissue. Confocal imaging was performed with a commercial microscopy system (SP5, Leica) equipped with a 20x objective. Excitation of the fluorophore was achieved at 561 nm (DPSS laser), and emission was detected from 600 to 700 nm.

### 2.3. Quantitative Analysis of Gene Expression

Total RNA was isolated from healthy and inflamed colon tissue, APCmin, and AOM + DSS tumors using RNeasy columns (Qiagen). cDNA was generated with the iScript cDNA synthesis kit (Bio-Rad Laboratories). Quantitative real-time PCR analysis for COX-2 and HPRT was conducted with QuantiTect Primer assays (Qiagen) and QuantiTect Sybr Green (Qiagen). Gene expression was calculated relative to the house-keeping gene HPRT using the ΔΔCt algorithm. 

### 2.4. Statistical Analysis

Data from qPCR analysis were compared with the Kruskal-Wallis test followed by Dunn's test for nonparametric samples with GraphPad Prism v 5.00.

## 3. Results

**Figure 1:**
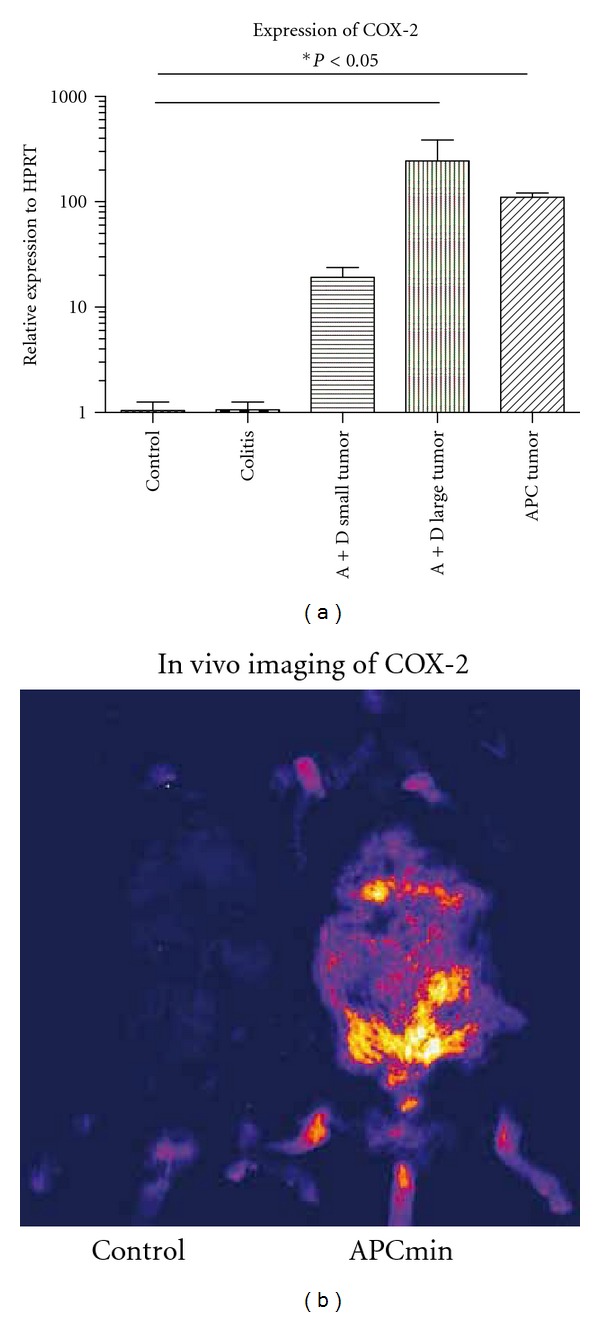
Expression of COX-2 in murine models of colitis, sporadic, and colitis-associated colorectal cancer. (a) Quantitative real-time PCR of COX-2 mRNA levels in control and inflamed colon tissue, small and large AOM + DSS induced tumors, and tumors of APCmin mice. Data are mean SEM. *N* = 3–5 per group. (b) Multispectral in vivo fluorescence imaging following the injection of a COX-2-specific fluorescence probe. One representative image of an APCmin mouse in comparison to a control animal is shown.

In line with data from the literature, COX-2 expression was significantly increased in large tumors of AOM + DSS-treated mice or APC mice (Figure 1(a)). In contrast, COX-2 mRNA levels in the inflamed colon tissue of AOM + DSS-treated animals were comparable to control tissues. 

In vivo full body fluorescence imaging of tumor-bearing mice following the injection of the COX-2 probe revealed a strong fluorescence in the lower abdominal region of these animals corresponding to the location of intestinal tumors of these mice (Figure 1(b)). Multispectral analysis of this signal verified the specific detection of the probe in comparison to autofluorescence.

**Figure 2:**
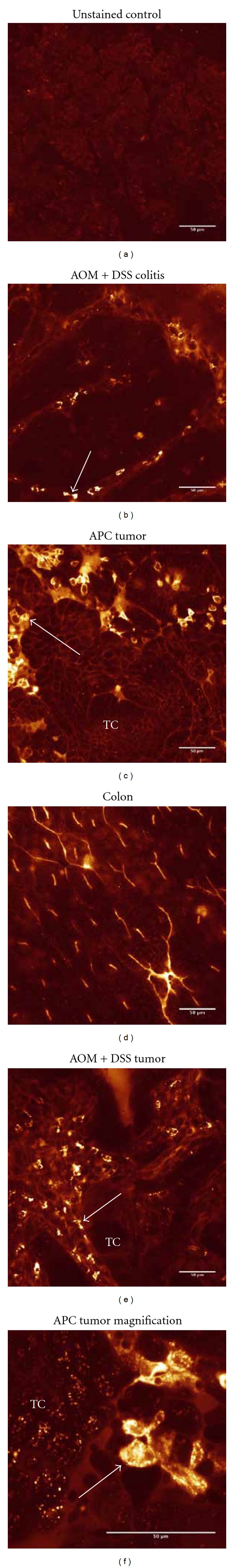
Confocal imaging of COX-2 expression. Confocal microscopy of COX-2 expression in unprocessed tissue specimens including healthy control tissue, inflamed colon tissue from AOM + DSS-treated animals and tumor tissue from AOM + DSS-treated animals and APCmin mice. The white bar represents 50 *μ*m. The white arrows indicate individual cells in the stroma of inflamed colon or tumor tissue with high COX-2 expression. TC: tumor cells.

Confocal imaging showed large amounts of infiltrating cells in the tumor stroma or at the tumor margin with a high COX-2 expression (Figure 2). Whereas nearly no COX-2 expressing cells were visible in healthy colon specimens, some COX-2 positive cells could be detected in the lamina propria of inflamed non-neoplastic colon tissue of AOM + DSS-treated mice. Interestingly, higher resolution imaging of tumor samples showed a weak expression of COX-2 in tumors epithelial cells in addition to the strong signal in stroma cells.

## 4. Discussion

Molecular imaging holds fascinating opportunities for the selective detection of specific cells and tissues by targeting unique markers of a particular disease of interest, such as inflammation or cancer. A potential target—in line with this basic concept—is COX-2 with a specific upregulation in premalignant and tumorous tissue and only very low expression in normal mucosa. In this preliminary, study we were able to specifically visualize high levels of COX-2 activity in mouse models of colitis-associated and sporadic CRC using confocal microscopy. Images obtained were comparable with immunohistology providing a subcellular resolution of COX-2 expression. Since endomicroscopy with similar scanners is already available for clinical application [18], molecular-targeted imaging of COX-2 could potentially be transferred into endoscopic routine.

In this regard, the endomicroscopic detection of COX-2 could serve two targets: (1) *the early detection of intestinal neoplasia and *(2) *a decision for chemoprevention.*

 (1) Regarding the early detection of intestinal neoplasia, the molecular detection of COX-2 expression could be a helpful marker during endoscopy. Due to the specific upregulation of COX-2 in tumor tissue [9], targeted biopsies could be taken in areas that show an increased number of COX-2 positive cells. In fact, COX-2 expression has been shown to be upregulated not only in colorectal carcinoma, but also in early dysplastic lesions such as aberrant crypt foci or sporadic polyps [19–21]. Therefore, the analysis of COX-2 mRNA levels in feces has previously been proposed as a screening test for CRC [22]. However, large clinical trials further supporting the analysis of COX-2 expression as a predictive marker for CRC are missing so far. Of note, some subtypes of CRC, such as CRC with a defective mismatch repair (MMR) system, are not associated with an increased COX-2 expression and could be missed with COX-2-dependent screening tests [23].

 (2) Despite the growing evidence supporting a chemopreventive effect of aspirin against CRC development, the general use of this therapeutic cannot be recommended due to the known side effects as discussed above. Interestingly, Chan et al. could show in a landmark study that the preventive effect of aspirin is only evident in patients that have an increased COX-2 expression [24]. Therefore, the endomicroscopic detection of COX-2 overexpression during surveillance endoscopy could identify patients with an increased risk for colorectal cancer development that could benefit from aspirin intake. As we have shown the technical feasibility for this approach in this paper, it would be interesting to evaluate this approach in a clinical study.

## 5. Conclusions

The data of this study clearly propose that molecular imaging of COX-2 expression in sporadic and colitis-associated cancer is possible with CLE. This technique could improve the detection of preneoplastic lesions during surveillance endoscopy or enable the identification of patients that would benefit from aspirin treatment as a preventive strategy against CRC development.
